# The Biological Functions of Non-coding RNAs: From a Line to a Circle

**DOI:** 10.15190/d.2015.40

**Published:** 2015-09-30

**Authors:** Nan Wu, Burton B. Yang

**Affiliations:** Sunnybrook Research Institute, Sunnybrook Health Sciences Centre, Toronto, Canada; Department of Laboratory Medicine and Pathobiology, University of Toronto, Toronto, Canada

**Keywords:** non-coding RNA, microRNA, long-non coding RNA, circular RNA, biological functions

## Abstract

Non-coding RNAs have gained increasing attention, as their physiological and pathological functions are being gradually uncovered. MicroRNAs are the most well-studied ncRNAs, which play essential roles in translational repression and mRNA degradation. In contrast, long non-coding RNAs are distinguished from other small/short non-coding RNAs by length and regulate chromatin remodeling, gene transcription and posttranscriptional modifications. Recently, circular RNAs have emerged as endogenous, abundant, conserved and stable in mammalian cells. It has been demonstrated that circular RNAs can function as miRNA sponges. Other possible biological functions of circular RNAs are still under investigation. In this review, the biogenesis and biological functions of the three major types of ncRNAs, including miRNAs, lncRNAs and circRNAs, are overviewed. In addition, the role of ncRNAs in human diseases and potential clinical applications of ncRNAs are discussed.

## SUMMARY

1. *Introduction*
2. *Biogenesis and functions of non-coding RNAs*
  - *2.1 MicroRNA*
  - *2.2 Long non-coding RNA*
  - *2.3 Circular RNA*
3. *Interaction between different non-coding RNAs*
  - *3.1 CircRNAs function as miRNA sponges*
  - *3.2 The interaction of pseudogene and non-coding transcripts with miRNAs*
4. *Non-coding RNA and diseases*
  - *4.1 MicroRNAs and diseases*
  - *4.2 Long non-coding RNAs and diseases*
  - *4.3 Circular RNAs and diseases*
5. *Future perspectives*

## 1. Introduction

In the human genome, only 1.5%-2% genes are protein-coding genes^1,2^. The vast majorities are known as non-protein coding genes, and have attracted increasing attention as their importance in physiological and pathological functions are being reported. Among the initial functional non-coding genes reported was a class of small non-coding RNA (ncRNA) called microRNAs (miRNA)^3^. Thereafter, other non-coding RNAs, such as PIWI-interacting RNAs (pi-RNAs), small nucleolar RNAs (snoRNAs), long non-coding RNAs (lncRNAs), and pseudogene were discovered and demonstrated to play roles in various development and disease processes^4-10^. Recently, the important functions of another class of ncRNAs, circular RNAs (circRNAs) has been illuminated, nearly 40 years following the initial characterization of circRNAs^11,12^.

Previously, ncRNAs were categorized according to their length. Although there is no clear borderline between classes, ncRNAs are broadly divided into short/small, mid-size, and long ncRNA^13^. The short ncRNAs includes miRNAs, piRNAs and tiRNAs. The snoRNAs belongs to mid-size RNAs. The large intergenic non-coding RNAs (lincRNAs) and the transcribed ultraconserved regions (T-UCR) with the size over 200bp are regarded as long ncRNAs^13^. Due to the special structure, circRNAs are classified either as an independent type of ncRNAs or as a class of long ncRNAs^14^.

In this review, we will give an overview of the biological functions of the three major types of ncRNAs, including miRNAs, lncRNAs and circRNAs. The biogenesis of these ncRNAs, especially circRNAs will also be briefly summarized. In addition, we will highlight the role of ncRNAs in the development of human diseases. Finally, we will bring to a discussion the existing and potential clinical application of ncRNAs.

## 2. Biogenesis and functions of non-coding RNAs

### 2.1 MicroRNA

MicroRNAs (miRNAs) are among the most well-studied ncRNAs. These are small, single-stranded RNAs of 18-24 nucleotides in length, that repress the transcription of their target genes^3,15,16^. It has been estimated that miRNAs regulate the translation of over 60% of protein-coding RNAs^16,17^. By controlling the majority of protein coding genes, miRNA have now been acknowledged for their essential roles in a range of physiological and pathological processes^18,19^.**

#### 2.1.1 Biogenesis of miRNA

**Figure 1:**
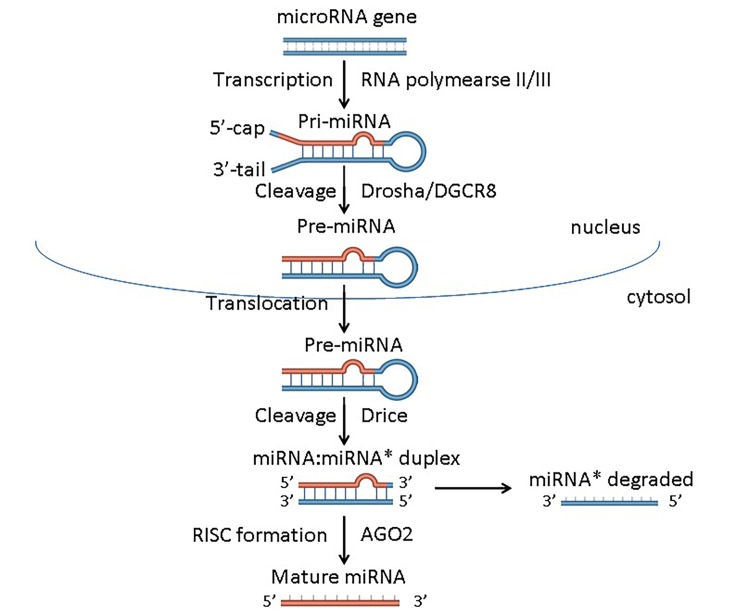
Figure 1. The biogenesis of microRNA The miRNA is initially transcribed by RNA Polymerase II (RNA Pol II) or RNA Pol III as pri-miRNA, then processed into pre-miRNA by Drosha/DGCR8 microprocessor complex. After transported from the nucleus to the cytoplasm, pre-miRNA is further processed by Dicer, releasing a small RNA duplex which is bound by an AGO protein and forms a complex with RISC. Then, the miRNA is regarded as mature miRNA. Abbreviations: pri-miRNA, primary miRNA; pre-miRNA, precursor miRNA; DGCR8, DiGeorge syndrome critical region 8; AGO, Argonaute; RISC, RNA-induced silencing complex.

Primary miRNAs (pri-miRNAs) are mostly transcribed by RNA Polymerase II (RNA Pol II), and RNA Pol III has been implicated in miRNA transcription in some viruses^20,21^. Following transcription, pri-miRNAs undergo nuclear processing by the Drosha/DGCR8 microprocessor complex, followed by export into the cytoplasm as pre-miRNAs complexed with exportin5 and RAN· GTP^22-25^. In the cytoplasm, the pre-miRNAs are further processed by Dicer, releasing a small RNA duplex^26-28^. Following Dicer cleavage, the small RNA duplex is subsequently bound by an Argonaute (AGO) protein, and forms a complex known as the RNA induced silencing complex (RISC)^29-31^. After assembly into RISC, the miRNAs are regarded as mature miRNAs which target the 3’-untranslated regions (3’UTR) of mRNAs. This leads to post-transcriptional repression via destabilizing mRNA and inhibiting translation initiation^17,32^. The process of miRNA biogenesis is summarized in **Figure 1**. In addition to the canonical pathway, a minor portion of miRNAs are generated by alternative pathways. The biogenesis of miR-451, for example, bypasses the microprocessor or Dicer^33-36^. The maturation of miR-451 is independent of Dicer because the precursor of miR-451 (pre-miR-451) which is cleaved by Dosha/DGCR8 is too short (~18 bp) to be a Dicer substrate. Alternatively, the pre-miR-451 is directly incorporated into AGO2 followed by spliced on the 3’ hairpin arm and further resected to generate mature miR-451^35,36^. In addition, the biogenesis of miRNAs can be regulated by transcription factors, such as p53, MYC, ZEB1 and ZEB2, and by epigenetic modification (e.g. methylation, adenylation)^37-39^.

#### 2.1.2 Functions of miRNA

As mentioned above, miRNAs form base pairs with complementary sequences on the 3’UTRs of the target mRNAs resulting in RNA silencing at post-transcriptional levels^40^. miRNAs perform their gene silencing functions via repressing translation, deadenylation and destabilizing mRNAs.

**Translational repression**

Translational repression may occur through four different mechanisms: inhibition of translation initiation, suppression of translation elongation, degradation of co-translational protein and acceleration of translation termination.

Pillai *et al*. first reported that miRNAs could inhibit the initiation of translation^41^. In this study as well as two other studies, it was demonstrated that miRNAs mediated repression of mRNA translation, and that this required the 5' cap structure and/or the 3' poly(A) tail^41-43^. A later *in vitro* study demonstrated that endogenous let-7 miRNAs inhibited the translation initiation by targeting the m^7^G-cap–recognition process^44^. Furthermore, another study demonstrated that Ago proteins could compete with eIF4E for cap binding, leading to the repression of translation initiation^45^. These studies all suggested that miRNAs targeted an early step of translation initiation.

Before these function of miRNAs on the inhibition of translation initiation were characterized, miRNAs were thought to suppress translation at the post-initiation stage. The evidence was found in both the *C. elegans* and mammalian cells^46-48^. In *C. elegans*, lin-4 miRNA repressed the translation of the lin‑14 and lin‑28 mRNAs. The lin‑14 and lin‑28 mRNAs could be detected in polysomes after the protein translation was repressed^47,48^. Subsequent studies in mammalian cells supported these findings in *C. elegans, *by providing evidence that miRNAs caused ribosome drop-off and silenced translation independent of the cap structure, indicating that miRNAs induced a premature termination of translation^48^. Nottrott *et al* using nascent polypeptide coimmunoprecipitation experiments demonstrated that human let-7a miRNA and mRNA associated in polysomes resulting in a degradation of the newly synthesized polypeptide^49^. These studies suggested that the repression of translation by miRNA could occur after translation initiation.

**mRNA deadenylation and degradation**

Other than directly regulating translation, miRNAs also can induce target mRNA degradation in both *C. elegans* and metazoans^32,50^. Studies showed that if specific miRNAs were transfected into cultured cells, the abundance of mRNAs which contained the complementary binding sites was decreased^32,51,52^. Vice versa, depletion of a miRNA or the essential components of the miRNA processing pathway, such as Dicer or AGO proteins resulted in a corresponding increase of the target mRNAs^51-53^. In miRNA directed mRNA degradation, deadenylation of the mRNAs, a widespread consequence of miRNA regulation, occurs before they undergo 5’-3’ mRNA decay pathway^54,55^.

### 2.2 Long non-coding RNA

Long non-coding RNAs (lncRNAs) are primary spliced non-protein-coding RNA transcripts that are longer than 200 nucleotides in length, and do not fit into known classes of non-coding RNAs^9,56^. The number of lncRNAs ranges from 10,000 to greater than 200,000, which indicates that lncRNA may make up the largest portion of the mammalian non-coding transcriptome^9,57^. Increasing evidence indicates that lncRNAs are crucial regulators of numerous biological pathways^8,55^.

#### 2.2.1 Biogenesis of lncRNA

LncRNAs can be transcribed from intergenic regions (lincRNAs), promoter regions (promoter-associated lncRNAs), introns of annotated genes (long intronic ncRNAs), the opposite strand of mRNAs (antisense lncRNAs), or pseudogenes^58-61^. Like coding genes, lncRNAs are transcribed by RNA polymerase II and then undergo post-transcriptional processing, including 5’capping, alternative splicing, RNA editing, and polyadenylation . However, unlike coding RNAs, cytoplasmic lncRNAs generally do not encode proteins^62^.

#### 2.2.2 Functions of lncRNA

**Epigenetic regulation and chromatin remodeling**

Long ncRNAs can mediate epigenetic changes as recruiters, tethers or scaffolds for chromatin remodeling. For example, Hox transcript antisense RNA (HOTAIR) represses the transcription of the HOXD in *trans* by inducing a repressive chromatin state through interaction with the Polycomb chromatin remodeling complex (PRC2)^63^. Similarly, a 17kb lncRNA, Xist and a related transcript called RepA also recruit PRC2^64^. Other than PRC2, HOTAIR can also bind LSD1/CoREST/REST complex that demethylates histone H3K4 to serve as a scaffold^65^. In addition, the imprinted lncRNA, named Air, also interacts with H3K9 methyltransferase G9a to epigenetically silence transcription^66^. A very recent study has identified another lncRNA, named myosin heavy-chain-associated RNA transcripts (Mhrt), which can bind to the helicase domain of Brg1 and sequester Brg1 from its genomic DNA targets to prevent chromatin remodeling^67^.

**Transcriptional regulation**

Long ncRNAs regulate gene transcription through a diverse set of mechanisms. Long ncRNAs can directly regulate gene transcription by acting as a decoy for transcription factors, transcriptional coregulators, or Pol II inhibitors. Some lncRNAs bind to certain RNA-binding proteins to silence gene expression. The ncRNA cyclin D1 (CCND1) can negatively regulate the parent CCND1 transcription by binding with and modulating the activities of the RNA-binding protein TLS, that subsequently suppress the histone acetyltransferase activities of CREB binding protein and p300^68^. A new identified lncRNA lnc-DC, which is expressed in human conventional dendritic cells, can bind directly to transcription factor STAT3 in the cytoplasm and promote STAT3 phosphorylation, via preventing STAT3 dephosphorylation by SHP1^69^. In addition to acting through RNA-binding proteins, lncRNAs can directly inhibit Pol II activity. Two lncRNAs in mammalian cells, mouse B2 RNA and human Alu RNA, both prevent Pol II from establishing interactions with the promoter during closed complex formation, leading to a disruption of these interactions associated with transcriptional repression^70,71^.

**Post-transcriptional regulation**

Long ncRNAs may also play a role in the regulation of mRNA degradation, protein stability and translation. The long ncRNA HOTAIR enhances Plk1-dependent ubiquitination of SUZ12 and ZNF198, two transcription repression factors, and significantly reduce SUZ12 and ZNF198 stability^72^. Another lncRNA, FAL1, has been demonstrated to be associated with the epigenetic repressor Polycomb complex protein, BMI1 and regulates its stability in order to modulate the transcription of a large set of genes that are related with cell cycle and apoptosis^73^. A long intergenic noncoding RNA (lincRNA) homologous to the mouse plasmacytoma variant translocation gene (PVT1) is reported to control levels of MYC through regulation of the MYC protein stability and consequently cooperate to promote cancer cell proliferation^74,75^. In addition to controlling mRNA and protein stability, lncRNAs also exert their effects at the level of translational regulation. Our previous study has demonstrated that tumour suppressor candidate-2 pseudogenes (TUSC2P) interact with several miRNAs resulting in increased translation of TUSC2^4^. Moreover, it has been reported that lincRNA-p21 associates with JunB, CTNNB1 and b-catenin mRNAs, selectively altering their translation via the translation repressor Rck^76^.

### 2.3. Circular RNA

Circular RNAs (circRNA) were first characterized in viruses in the 1970s^77^. However, due to their unique structure and limitations of detection techniques, the roles of circRNA have been underestimated over the past several decades. Last year, large amounts of endogenous circRNA were revealed in mammalian cells with the improvement of computational techniques and higher throughput sequencing methods^12,78-80^. Unlike linear RNA, circRNAs form a covalently closed continuous loop^81^ and its expression levels are not correlated with those of their linear isoforms^79^. Recently, circRNA have been identified as a type of non-coding RNA. CircRNAs now have been categorized into three types: exonic, circular intronic and retained-intron circRNAs, based on their origins and sequences^12,14,78-82^.

#### 2.3.1 Biogenesis and properties of circRNA

**Figure 2:**
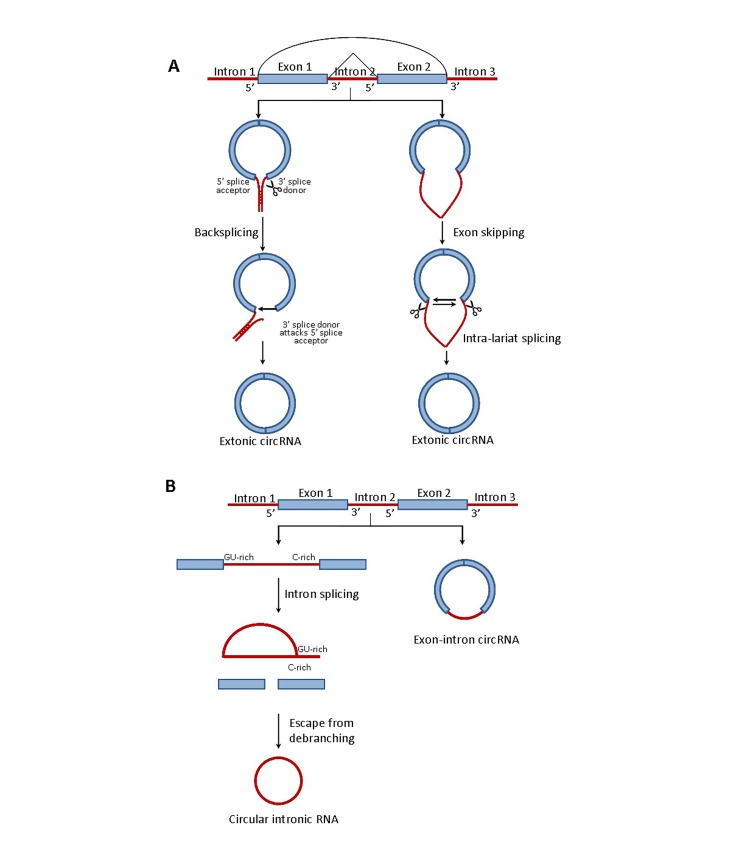
Figure 2. The biogenesis of circRNA **A.** The biogenesis of extronic circRNA. The extronic circRNA can be formed via either backsplicing or extron skipping. Backsplicing is a downstream splicer donor pairs with unspliced upstream splice acceptor and the invervening RNA is circularized. Extron skipping leads to an exon-containing lariat, which could be further internally spliced to an exonic circular RNA molecule. **B.** The biogenesis of circular intronic RNA and extron-intro circRNA. The biogenesis of circular intronic RNAs (ciRNAs) depends on a consensus motif containing a GU-rich element near the 5’ splice site and a C-rich element close to the branchpoint site. These motifs lead to a failure of debranching, which escape the lariat introns from branching and facilitate the formation of ciRNAs. The exon-intron circRNAs are generated from circularized exons with introns retained between exons.

The biogenesis of circRNAs is still under investigation. Most circRNA molecules in eukaryotic cells are produced via backsplicing by the spliceosomal machinery which joins a splice donor to an upstream splice acceptor^81,83^. Originally, the majority of circRNAs were regarded as the by-products of mis-splicing. However, a series of recent studies indicated that the formation of circRNAs is fully regulated. Over 250,000 circRNAs were identified from 14.4% of actively transcribed genes in human fibroblasts and these circRNAs were demonstrated to be stable and conserved^78^. Generally, the biogenesis of circRNA is as shown in**Figure 2**. A recent report demonstrated that exon circularization required flanking intronic complementary sequences. In addition, alternative circularization, resulting from the competition between the flanking introns or individual introns, may lead to a production of multiple circRNA transcripts from a single gene^81^. In accordance with this study, Ashwal-Fluss, *et al*. also elucidated that the production rate of circRNAs is mainly determined by flanking introns and precursor mRNA splicing, competing with circularization of exons^83^. In addition, short intronic repeats in the flanking introns were demonstrated to be critical for circularization^84^. These results all suggest that a close proximity of exon-intron junctions resulting from complementary base pairing of inverted repeats in the flanking introns facilitate the biogenesis of circRNAs. On the other hand, circRNAs have also been suggested to be formed by exon skipping^75,85^. Exon skipping leads to an exon-containing lariat, which could be further internally spliced to an exonic circular RNA molecule^78,85^.

In contrast to the circRNAs from exon back-splicing, the circular intronic RNAs (ciRNAs) are synthesized from the Group I or Group II introns which are excised from precursor RNAs. The previous studies have demonstrated that the full-length ciRNAs from Group I introns are generated via autocatalytic ribozyme action in *Tetrahymena*.

In brief, the circularization of RNAs is initiated by sequential site-specific hydrolysis followed by a transesterification action requiring guanosine as cofactor^86^. Another recent study has found that Group I intron could perform 3’, 5’ ligation to form circRNA and the exogenous guanosine cofactor is added during the steps of self-splicing^87^. The formation of ciRNAs which are from Group II introns depend on a consensus motif containing a 7 nt GU-rich element near the 5’ splice site and an 11 nt C-rich element close to the branchpoint site^14^. These motifs lead to a failure of debranching, which escape the lariat introns from branching and facilitate the formation of ciRNAs^14^. Recently, another subclass of circRNAs, named exon-intron circRNAs, have been highlighted. Exon-intron circRNAs are generated from circularized exons with introns retained between exons^88^. Both the ciRNAs and exon-intron circRNAs have been suggested to be involved in Pol II transcription^14,88^. The role of these circRNAs on the regulation of transcription will be discussed in the following section.

The biogenesis of circRNAs can be regulated by some RNA-binding proteins. The second exon of the splicing factor *muscleblind* (MBL/MBNL1) has been reported to promote the circularization of its own exons during circMbl biogenesis, by strongly and specifically binding to the conserved MBL binding sites in circMbl and its flanking introns^83^. Another RNA binding protein, Quaking (QKI), has been demonstrated to be a major regulator of circRNA biogenesis in epithelial-mesenchymal transition (EMT). Unlike MBL, QKI does not regulate the formation of its own circRNA. Instead, knockdown of QKI reduces the abundance of 105 out of 300 circRNAs by more than 2-fold in mesHMLE cells^89^. In accordance, a very recent study reported that a conserved RNA-editing enzyme, ADAR, may also be involved in circRNA biogenesis^90^.

#### 2.3.2 Functions of circRNA

A large amount of circRNAs have been identified using genome-wide analysis^78-80^. Identification of the essential physiological roles of circRNAs has become a hotspot of ncRNA research. It may be possible that circRNAs function as miRNA sponges, thereby regulating gene expression at the transcriptional and translational levels.

** **Regulation of transcription**

Among the first identified function of circRNA was that of a miRNA sponge, through which circRNAs indirectly regulated the transcription of target mRNA. In this review, the interaction of circRNAs and miRNAs will be summarized in the later section. Other than serving as a miRNA sponge however, circRNAs can also form base-pairs with RNA or sequester RNA-binding proteins^12,91^. The ciRNAs contain very few miRNA binding sites and act mainly in the nucleus. Some ciRNAs accumulate to the site of transcription bind with RNA Pol II and act as a positive regulator of RNA Pol II transcription, by which they control the expression of their parent genes^14^. Interestingly, besides these transcription sites, ciRNAs may also play a *trans* regulatory effect for the other target genes^14^. Exon-intron circRNAs have been demonstrated to be predominantly localized in the nucleus and exert *cis *regulatory effect on mRNA via interaction with Pol II, U1 snRNP and gene promoters^88^.

**Regulation of translation**

It is possible that an endogenous circRNA with an internal ribosome entry site (IRES) and a start codon could undergo translation. However, there remains no evidence that ATG-containing exonic circRNAs undergo translation in eukaryotes. The only natural circRNA which can encode a protein is circular hepatitis d in the hepatitis B virus^92^.

## 3. Interaction between different non-coding RNAs

### 3.1 CircRNAs function as miRNA sponges

In 2013, two groups published their findings regarding circular CDR1 antisense (CDR1as) or circular transcript ciRS-7, and the ability to suppress miR-7 activity^11,12^. There are more than 70 conventional seed–target regions for miR-7 located on the CDR1as/ciRS-7. In addition, CDR1as/ciRS-7 is densely associated with AGO proteins in a miR-7-dependent manner^11,12^. Knockdown of CDR1as/ciRS-7 leads to a decreased expression of miR-7 target genes. Overexpression of CDR1as/ciRS-7 results in the phenotypes that are similar to miRNA inhibition^12^. Besides CDR1as, circular sry (sex-determining region Y) can also serve as miRNA sponges for miR-138, indicating that the functions of circRNA as miRNA sponges may be observed in multiple biological contexts^11^. In a recent study, the function of circRNA as a miRNA sponge was further confirmed. During the development of esophageal squamous cell carcinoma (ESCC), circITCH serves as a sponge for miR-7, miR-17 and miR-214, thereby increasing levels of the ITCH mRNA, inhibiting the Wnt/β-catenin pathway^93^. Interestingly, another study has recently argued that most circRNAs are low-abundance and have less miRNA binding site than CDR1as. It remains to be seen whether other circRNAs function as miRNA sponges, as effectively as CDR1as^94^. Whether more circRNAs act as miRNA sponges warrants further investigation.

### 3.2 The interaction of pseudogene and non-coding transcripts with miRNAs**

Pseudogenes are a subclass of lncRNAs. Our previous studies have demonstrated that the pseudogene TUSC2P can interact with endogenous miRNAs including miR-17, miR-93, miR-299-3p, miR-520a, miR-608 and miR-661, resulting in increased translation of TUSC2, TIMP2 and TIMP3^4^. Another lncRNA PTEN pseudogene, PTENP1, serves as a tumour suppressive, by acting as a decoy for PTEN-targeting miR-17, miR-21, miR-214, miR-19 and miR-26 families^10^. In addition, the non-coding fragments of mRNAs can also function as sponge to regulate miRNA activities. For example, HMGA2 can function as a competing endogenous RNA to regulate miRNA activity^95^. The 3’-untranslated regions of versican^96-98^, CD44^99,100^, and nephronetin^101,102^ can bind to a number of endogenous miRNAs and function as sponges to modulate miRNA activities.

## 4. Non-coding RNAs and diseases

### 4.1 MicroRNAs and diseases

Following over a decade of research, miRNAs are now widely acknowledged to play crucial roles in carcinogenesis, as either tumor suppressors or oncogenes. Many studies are devoted to investigate have elucidated the diverse roles of miRNAs in the initiation and development of cancer. For example, our previous studies have demonstrated that miR-17 exerts dual effects on tumorigenesis. In hepatocarcinoma and prostate cancers, miR-17 promotes tumor growth and invasion by inhibiting the expression of tumor suppressors, such as PTEN or TIMP3^103,104^. In breast cancer, miR-24, miR-378, miR-93 and miR-199 enhances cell growth, survival, invasion, angiogenesis and metastasis by targeting different tumor suppressors^105-108^. In melanoma cells, miR-17 represses growth by targeting STAT3 and stimulating downstream host immune responses^109^. In addition, miR-17 also reduces cell adhesion, migration and proliferation in different normal or cancer cells^110^. Even in the same tumor cells, miR-17 shows dual roles in cell growth: it reduces the tumor proliferation but prolongs tumor cell survival and induces angiogenesis^111^. In accordance, other studies have also identified the dual roles of miR-17 or other miRNAs in other cancer types^112,113^.

**Figure 3:**
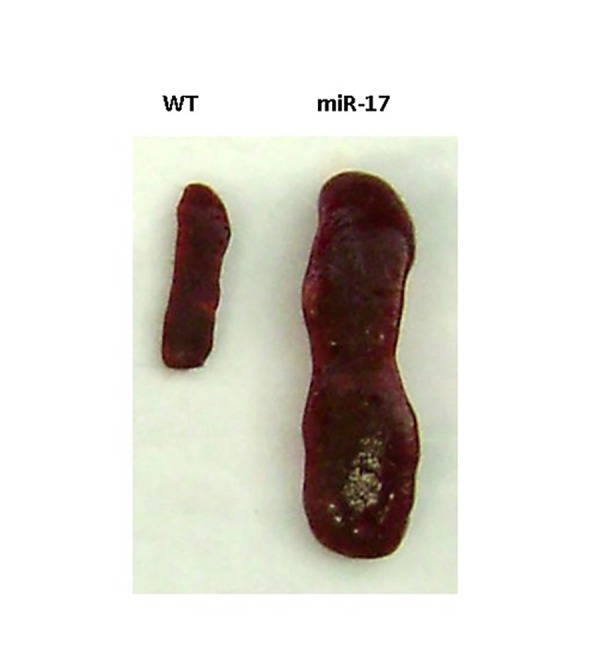
Figure 3. Role of miR-17 in organ size The spleen size of miR-17 transgenic mice (miR-17) is significantly increased compared to wide type mice (WT).

Other than their roles in cancer, miRNAs are also involved in a variety of human diseases and biological processes. Our recent work shows that miRNAs, including miR-17 and miR-378 are important to wound healing, senescence as well as lipid metabolism^114-117^. We’ve also found that miR-17 controls the organ development. Transgenic mice overexpressing miR-17 showed growth retardation and smaller organs^110^. In the older mice, spontaneous hepatocellular carcinoma developed^103^. Further examination of the mice showed that larger spleens were observed in miR-17 transgenic mice compared to the wildtype mice (**Figure 3**). This suggests inflammation occurred in the miR-17 transgenic mice. The molecular mechanism awaits further investigation.

In addition, a number of large scale studies have identified associations between miRNAs and a range of diseases^118^. Several databases have been created to help the researches to understand the association between miRNAs and human diseases, such as human miRNA-associated disease database (http://cmbi.bjmu.edu.cn/hmdd), miR2Disease (http://www.mir2disease.org/) and PhenomiR (http://mips.helmholtz-muenchen.de/ phenomir)^119-121^. The investigation of the relationship between miRNA and human disease will help to identify more biomarkers and potential therapeutic targets.

### 4.2 Long non-coding RNAs and diseases

There is an increasing interest in researching the potential involvement of lncRNAs in diseases, due to their diverse functions in differentiation and developmental processes. lncRNAs appear to be new players in cancer and their potential roles have been demonstrated to involve in both oncogenic and tumor suppressive pathways^122-124^. It has been demonstrated that the aberrant expression of lncRNAs are associated with a variety of human tumors, such as breast, ovarian, prostate, and hepatocellular carcinoma^10,72-74,125^. Our previous study found that the pseudogene TUSC2P could inhibit cell proliferation, survival, migration, invasion and colony formation, and increase tumour cell death in breast cancer cells, via regulating TUSC2P, TIMP2 and TIMP3^4^. Other than in cancer, lncRNAs may also play important roles in the development of neurodegenerative diseases (e.g. Alzheimer’s disease) and autoimmune disease (e.g. systemic lupus erythematosus)^126,127^. Similar to miRNAs, there are also useful database resources providing the putative or validated association of lncRNAs and diseases^128-130^, such as LncRNADisease database (http://cmbi.bjmu.edu.cn/lncrnadisease),lncRNAdb (http://www.lncrnadb.org/), and lncRNome (http://genome.igib.res.in/lncRNome), and other ones. Although all of the databases have certain limitations, these lncRNA databases help to delineate the relationships between lncRNA transcripts and their functions.

### 4.3 Circular RNAs and diseases

Due to its special structure and functions, circRNAs are considered to play critical roles in physiological and pathological processes and, therefore, may be involved in the initiation and development of certain diseases. To check the enrichment of genes associated with particular biological processes, a set of protein coding genes in the miRNA-circRNA interactome of diseases were analyzed using Gene Ontology (GO) enrichment analysis^131^. A database of disease-circRNA association was compiled in Circ2Traits, which was the first database of potential correlations between circular RNAs with human diseases^131^. In this study, it has been demonstrated that circRNAs are related to a variety of diseases, including gastric, esophageal, and prostate cancers, as well as neurodegenerative diseases like Parkinson's disease, Alzheimer's disease, Multiple sclerosis, and schizophrenia^131^.

**circRNA and neurological disorders**

circRNAs are highly enriched in the mammalian brain and *Drosophila* neural tissues^1,12,132,133^. On one hand, circRNAs, such as CDR1as/ciRS-7, could possibly be involved in diseases associated with miRNA owing to its function as a miRNA sponge^12^. For example, CiRS-7 is significantly decreased in the Alzheimer’s disease (AD) hippocampal CA1 samples compared to those in the age-matched controls^134^. Deficiency of ciRS-7 may result in an increase of miR-7 levels in AD brain cells, which is expected to downregulate the expression of miR-7 target genes relevant to AD^134^. On the other hand, circRNAs have been demonstrated to be upregulated during neuronal differentiation and development, and highly enriched in synapses in mouse and human brains^132^. In *Drosophila*, circRNAs also accumulate in the brain and increase with aging, which indicates the circRNAs may play a role in neural aging and have the potential to be an aging marker in neural system^133^.

**circRNA and cancer**

Based on a gene ontology (GO) enrichment analysis, Ghosal et al. established a global view of the potential association of circRNAs with cancer^131^. In this study, more than 60 of the circRNA which interacted with mRNAs were associated with breast and gastric cancers, and almost 200 of such circRNA-interacted mRNAs were correlated to cervical cancer. Further to this, some studies have begun to examine the role of circRNAs in specific cancers. Li P et al. identified that the circRNA hsa_circ_002059 was significantly downregulated in gastric cancer tissues compared with paired adjacent non-tumorous tissues, indicating this circRNA may hold promise as a potential biomarker for the diagnosis of gastric carcinoma^135^. In addition, another study has indicated that the circRNA abundance is low in colorectal cancer cell lines and cancer compared to normal tissues after detecting over 1,800 circular RNAs, suggesting a negative correlation of circular RNA abundance and proliferation^136^. In accordance with these studies, Li F, *et al.* also delineated that circITCH expression was low in ESCC compared to the peritumoral tissue^93^. In contrast, emerging data from our group has indicated that overexpression of some circRNAs could significantly promote tumorigenesis. Therefore, whether circRNAs are regarded as oncogenes or tumor suppressors will likely depend on the downstream target genes or proteins that are regulated by individual circRNAs.

## 5. Future perspectives

With the development of emerging computational, high throughput sequencing and biological techniques, we are able to further investigate and understand ncRNAs generated from different mechanisms. The RNAs previously thought as ‘junk’ RNA are now considered to be the treasures of the modern RNA research world. Only approximately 1.4% of the human genome is translated into proteins, whereas about 25% of mammalian genomes are predicted to be transcribed but not translated^1,2,56,137^, giving rise to a vast array of ncRNA which may play critical biological functions. Progressively, over the last four decades, researchers have found the structures of ncRNAs to range from short to long, and from linear to circular. Long ncRNAs and circular RNAs have been demonstrated to interact with miRNAs and exert a diverse range of biological functions. There remain large gaps in our understanding of ncRNAs, including the proportion and the range of ncRNA that are functional, and a mechanistic basis of these functions. The biogenesis pathways of these ncRNAs still warrants further study and is not clearly understood. As for miRNAs, which are the most studied ncRNAs, a cancer-targeted miRNA drug, MRX34 (a liposome-based miR-34 mimic), was recently launched into Phase I clinical trials in patients with advanced hepatocellular carcinoma in April 2013^138,139^. In addition, an LNA anti-miR against miR-122, was recently evaluated in Phase I and Phase IIa clinical trials for the treatment of hepatitis C virus^140,141^. The positive results from these clinical trials are encouraging, giving scientists, physicians and patients reason to further investigate the therapeutic potential of ncRNAs. Since lncRNAs and cirRNAs are more stable and specific for the target genes than miRNAs are, they may provide a new set of opportunities for clinical translation, following detailed preclinical examination of these ncRNAs.

- **Although non-coding RNAs do not encode proteins, they are essential in controlling the biological process associated with transcription, translation and/or post-transcriptional and post-translational regulation.**
- **LncRNAs and circRNAs interact with miRNAs and exert a diverse range of biological functions.**
- **Non-coding RNAs play critical roles in the initiation and progression of a variety of diseases.**
- **Recent clinical trials for the application of miRNA-based therapeutics in certain diseases are encouraging, which indicates the great potential for the bench-to-bedside translation of lncRNAs and circRNAs in the future.**
